# Quantitative Models for Prediction of Cumulative Trauma Disorders Applied to the Maquiladora Industry

**DOI:** 10.3390/ijerph18073830

**Published:** 2021-04-06

**Authors:** Melissa Airem Cázares-Manríquez, Claudia Camargo-Wilson, Ricardo Vardasca, Jorge Luis García-Alcaraz, Jesús Everardo Olguín-Tiznado, Juan Andrés López-Barreras, Blanca Rosa García-Rivera

**Affiliations:** 1 Faculty of Engineering, Arquitecture and Design, Autonomous University of Baja California, Ensenada BC 22860, Mexico; airem.cazares@uabc.edu.mx (M.A.C.-M.); ccamargo@uabc.edu.mx (C.C.-W.); jeol79@uabc.edu.mx (J.E.O.-T.); 2 Faculdade de Engenharia, Universidade do Porto, 4200-465 Porto, Portugal; ricardo.vardasca@fe.up.pt or; 3 INEGI, Universidade do Porto, 4200-465 Porto, Portugal; 4 ISLA Santarém, 2000-241 Santarém, Portugal; 5 Department of Industrial Engineering and Manufacturing, Autonomous University of Ciudad Juarez, Ciudad Juárez CHIH 32310, Mexico; 6 Faculty of Chemical Sciences and Engineering, Autonomous University of Baja California, Tijuana BC 22390, Mexico; jalopez@uabc.edu.mx; 7 Faculty of Administrative and Social Sciences, Autonomous University of Baja California, Tijuana BC 22390, Mexico; blanca_garcia@uabc.edu.mx

**Keywords:** age, blood pressure, body mass index, carpal tunnel syndrome, cumulative trauma disorder, heart rate, infrared thermography, respiratory rate, gender

## Abstract

Temperature gradient changes on the surface of the skin or in the middle of the body are signs of a disease. The aim of this study is to develop quantitative models for the prediction of cumulative trauma disorders (CTDs) arising from highly repetitive activities, considering risk factors, such as age, gender, body mass index (BMI), blood pressure (BP), respiratory rate (RR), and heart rate, to prevent injuries in manufacturing factory operators. This research involved 19 individuals from the area of sanding and 14 individuals from the area of tolex in manufacturing factories who had their vital signs and somatometry taken, as well as thermal images of their hands in the dorsal and palmar areas; an evaluation by the OCRA method was also applied. Factors such as BP and heart rate were determined to significantly influence the injuries, but no strong association with BMI was found. Quadratic regression models were developed, the estimates of which were adequately adjusted to the variable (R^2^ and R^2^ adjusted > 0.70). When integrating the factors of the OCRA method to the generated models, a better fit was obtained (R^2^ and adjusted R^2^ > 0.80). In conclusion, the participants who present levels out of the normal range in at least one of the factors have high probabilities of developing injuries in their wrists.

## 1. Introduction

Musculoskeletal system disorders (MSDs) refer to health problems in the locomotive system; that is, muscles, tendons, skeleton, cartilage, joints, ligaments, blood vessels, and tendons [1,2]. MSDs are a set of symptoms and injuries (inflammatory or degenerative) of the musculoskeletal system, and are related to the neck, back, and upper and lower extremities of the body [3]. MSDs are the most common occupational diseases in industry. They derive from various causes, and are divided into two categories: those caused by acute trauma, such as slips or falls, and those due to repetitive exposure to a type of physical activity, known as cumulative trauma disorders (CTDs), meaning that these injuries develop over time, which can be as long as weeks, months, or even years of propensity to repetitive stress, so that they are not due to a single temporary event, as is the case of the first category, but to various micro-traumas [1,2].

When MSDs are caused by work-related issues, they are called work-related musculoskeletal disorders (WMSDs) [4]. For years, studies have focused on ways to reduce WMSDs. General knowledge of the mechanisms and factors that cause the given ailments, among others, has enabled the development of a series of methods for risk occurrence and identification. WMSDs can be prevented through ergonomic interventions, including optimization of posture and working conditions, muscle and movement training, periodic work breaks, and load-dependent work management, through which the load on the musculoskeletal system can be reduced, thus providing workers with a longer working life [5].

Workers who suffer from this disorder may experience severe pain, which is reflected in a decrease in productivity and quality of work, and can even cause disability, which causes absenteeism from work and leads to increased costs for businesses and for the public health system [2]. Pain caused by musculoskeletal disorders is the second leading cause of disability [6], and, according to the International Labour Organization (ILO), it is estimated that occupational accidents and diseases cause the loss of 4% of the gross domestic product (GDP), or about $2.8 billion in direct and indirect costs.

MSDs occur in different areas of the body, caused by a variety of different types of tasks. In the upper extremities, such as the fingers, hands, wrists, arms, elbows, shoulders, and neck, MSDs can originate from repetitive or lasting static force, leading to tendinitis or nerve entrapment, such as carpal tunnel syndrome (CTS) [2,7,8,9]. CTS is due to compression of the median nerve inside the carpal tunnel, while flexor tendinitis causes compression of the median nerve by increased pressure in the carpal tunnel due to edema.

Currently, there are several methods that allow us to detect MSDs. For example, CTS is detected by means of palpation tests, such as the Phalen’s and Tinel’s tests, and electromyography. However, the use of thermal imaging may improve medical diagnosis [10].

Temperature gradient changes (decrease and increase) on the skin surface or in the middle of the body are indicators of disease, allowing the evaluation of changes in metabolism and blood flow, especially in a superficial layer of the skin [10,11,12,13,14,15]. Several studies indicate that the symmetry of the extremities and torso will not have a temperature difference on the two sides along a dermatome or thermatome of more than 0.30 °C, and of no more than 0.90 °C on the forearms [16]. The diagnosis of neuromuscular pathology by infrared thermography (IT) is based on the existence of thermal symmetry and asymmetry between normal and abnormal sites [17,18]. IT works by measuring the temperature distribution of a surface, which offers several advantages, because it is non-invasive, non-contact, non-radioactive, and painless, and the results are easy to reproduce (thermal imaging); it also has a low operating cost [10,19,20,21]. A broad range of research has proven the effectiveness of IT in diagnosing CTS [20,21].

Nowadays, CTS is a pathology of great interest in medical research, since it represents one of the greatest occupational health problems of any upper limb disorder [22], and yet, the etiology is not appropriately described [22,23].

Epidemiological studies have been undertaken to identify risk factors for CTS, and the results are contradictory. However, the most consistent factors have been being female [22,23,24,25,26,27,28,29], thirty years or older [22,23,25,26,28,29,30,31,32,33,34,35,36], having repetitive motor activity, and having a number of systemic diseases, such as diabetes mellitus [10], rheumatoid arthritis [37,38], and hypothyroidism [37,39].

Campillo & De la Vega [40] developed a predictive model for CTDs by using sensory thermography as the main tool. They sought to determine whether there is a relationship between temperature variability and CTD diagnosis and, at the same time, whether there is a gender difference regarding CTDs. However, the model does not explain the temperature variation over time well. In turn, Márquez Gómez [41] used traditional methods, such as RULA (Rapid Upper Limb Assessment) and OCRA (Occupational Repetitive Action), in combination with statistical techniques for the selection of significant predictor variables for the development of predictive models. Grieco [42] reported a logarithmic conversion of the relative exposure (OCRA) and injury indices, with which he constructed a simple linear regression model for risk prediction of WRMSDs. In the same context, Álvarez-Tello et al. [43] developed a predictive model using binary logistic regression and the items of the strain index questionnaire as predictor variables. The aim of this study is to develop quantitative predictive models that integrate risk factors for CTD, such as age, sex, BMI (body mass index), blood pressure (BP), respiratory rate (RR), and heart rate.

## 2. Materials and Methods

### 2.1. Recruitment and Selection of Participants

At first, the target company was approached to explain the purposes of the research and to request approval to apply the project by means of a document expressing the objectives, procedures, and analyses to be carried out, as well as estimated times. Once permission was obtained from the business authorities, the study was initiated based on the clinical procedures established by the company’s occupational health department.

Two production areas were assigned, the sanding and tolex areas, which had the highest records for wrist problems among the operators. In the sanding area, the activities of the operators consist of the sanding process of the body, neck, and edges of the wood product using orbital and edge sanders. On the other hand, in the tolex area, the cabinet subassembly and lining process is performed, which includes the activities of vinyl and fabric cutting, gluing, and stapling. Next, each of the areas was visited to learn about their production processes and to determine the experimental space. Afterwards, a questionnaire was given to each operator (a total of 39 persons), designed to select the participants of the study, obtaining their socio-demographic information and health conditions. This phase had an approximate duration of one month, due to the time restriction so as not to affect the daily production goals of the company.

Twenty-three questionnaires were applied in the sanding area and 16 in the tolex area. At the end of the recruitment process, 19 participants were selected from the sanding area (four persons were not selected due to disabilities and diabetes). Sixteen questionnaires were applied in the tolex area, and 14 participants were chosen (one person did not want to participate and another one had epilepsy). One woman and eighteen men participated in the sanding area (average age = 33 ± 9.7 years). Six women and eight men participated in the tolex area (average age = 35 ± 7.45 years). The experimental sample included a total of 33 people.

Then, the vital signs and somatometry of the chosen participants were recorded, including weight, height, body mass index (BMI), blood pressure (BP), heart rate, and respiratory rate (R.R). The sanding area showed an average BMI = 27 kg/m^2^, BP 78% normal, 13% high, and 9% low; 96% of the participants were right-handed, had an average heart rate of 77.43 beats per minute (BPM), and an RR of 17.63 breaths per minute. In the tolex area, an average BMI of 28.4 kg/m^2^, BP 75% normal, 12.5% high, and 12.5% low was recorded. All of the participants in this area were right-handed, and had an average heart rate of 94.18 BPM and an RR of 18.5 breaths per minute.

The selected subjects did not take drugs for the peripheral nervous system (vasodilator, antihypertensive) so as not to interrupt the sympathetic vasoconstrictive response and, therefore, affect their body temperature. Furthermore, they were asked to meet certain criteria in order to take the thermograms listed below. Data collection and thermograms at the company began in February 2019 and ended in June 2019.

**Figure 1:**
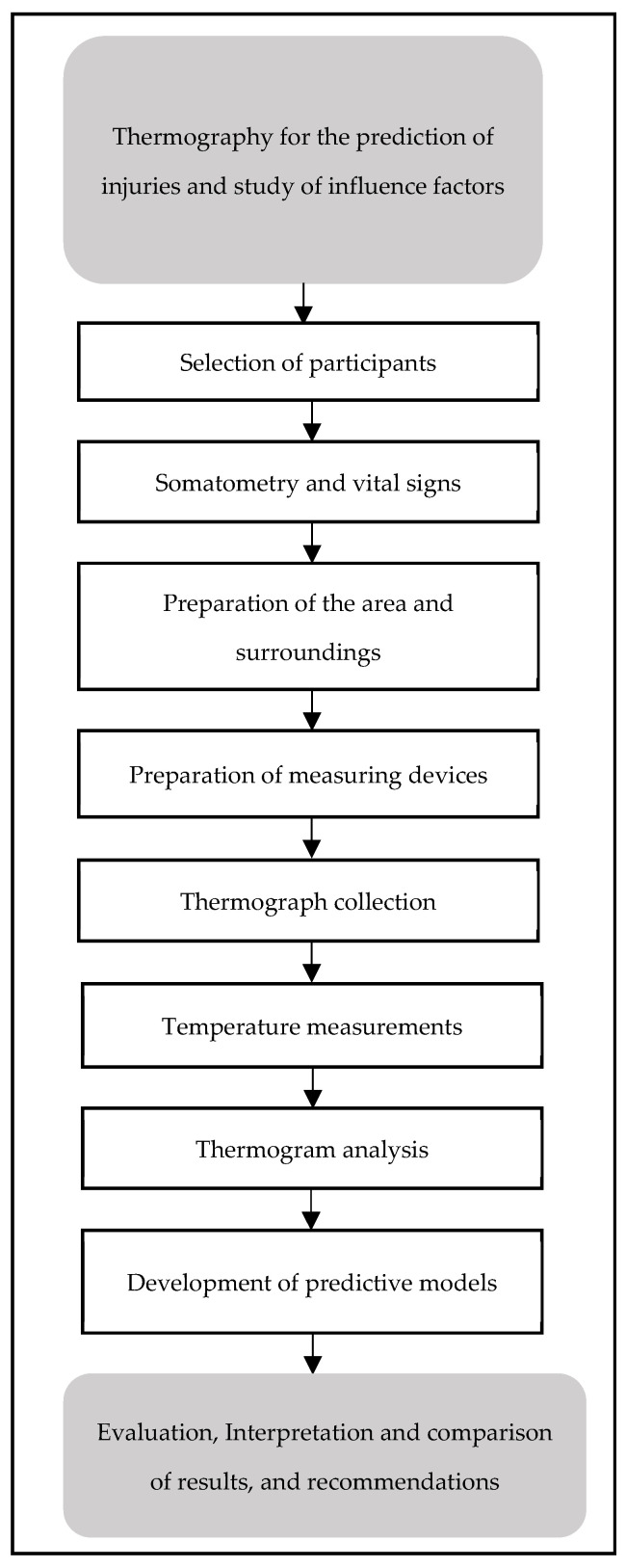
Measurement methodology flowchart.

A diagram with the measurement methodology appears in Figure 1.

### 2.2. Statement of Ethics

This study was conducted in accordance with the written consent granted by the company, which was provided verbally to all participants. The protocol was reviewed and approved by the ethics and bioethics committee of the postgraduate department of the Faculty of Engineering, Architecture, and Design of the Autonomous University of Baja California, according to the NOM-035-STPS-2018 Standard.

### 2.3. Preliminary Restrictions

Prior to taking the thermograms and in order to eliminate uncertainties in temperature measurements, the following restrictions were imposed on participants, based on the protocols of Glamorgan [44], Standard Procedures for Infrared Imaging in Medicine [45], and Design and Application of a Protocol for Acquiring and Processing Infrared Images from the Hands [46].

- Not to smoke in the hours prior to taking the images (12 hours).
- Not to drink alcoholic beverages in the hours prior to the exam (12 hours).
- Not to drink coffee or tea for several hours before the study (12 hours).
- Preferably, not to eat fatty foods before the analysis.

### 2.4. Experimental Protocol

#### 2.4.1. Environmental Conditions for the Study

To avoid vasomotion phenomena, the controlled temperature of the rooms assigned by each area was kept between 23–24 °C (+/−1 °C). Regarding humidity, its values oscillated between 50–60%, depending on the weather conditions of the region. On days when the humidity of the environment was high, a dehumidifier was required to reduce it to adequate levels. Within the space allocated for the recordings, air drafts on the subjects’ hands and lamps or domes above them were avoided during the taking of the thermal images. The participants were asked to uncover their forearm (if necessary), not to wear bracelets, rings, or wristbands, and to remove earrings, glasses, and caps.

#### 2.4.2. Thermographic Infrared Camera Implementation

**Figure 2:**
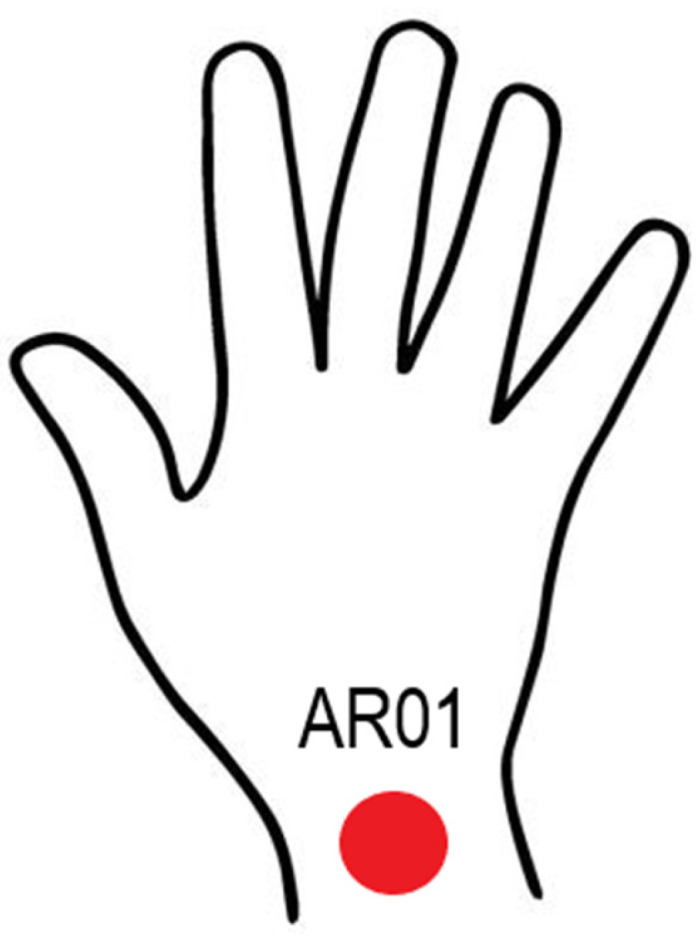
ROI (region of interest) taken for temperature analysis on the palms and back of the hand of the study subjects.

The IT camera used in this study was a FLIR ThermaCAM^TM^ E25 model, fabricated by FLIR Systems at Boston, MA, USA, with a resolution of 160 × 120 pixels, an accuracy of ±2 °C/±3.6 °C for ±2% of reading, and a spectral range of 7.5–13 μm. The camera was mounted on a tripod for better handling, with an emissivity of 0.98, as this is the average emissivity of human skin, and thus avoids errors in temperature measurement. Each time the infrared camera was used, the emissivity was set to this value. The chosen region (Figure 2) was taken for all participants. Before each shot, the camera was kept turned on for 15 minutes to maintain thermal equilibrium with its surroundings. The camera was placed perpendicular to the subject’s hand at a minimum distance of 0.601 m [46]. For this study, a distance of two meters was considered. It is worth mentioning that a black surface was placed as a background for the image, contributing to the improvement of the reading of the thermograms and reducing the surrounding noise.

#### 2.4.3. Handling of the Participants

Prior to the start of the test, each participant was checked for compliance with the requirements, that is, no caffeine, alcohol, vasodilator drugs, or smoking, to continue with the tests. For this purpose, they were given a reminder the day before the tests were to take place. The female menstrual cycle was considered. Nevertheless, none of the participants had their menstrual period during the intakes.

**Figure 3:**
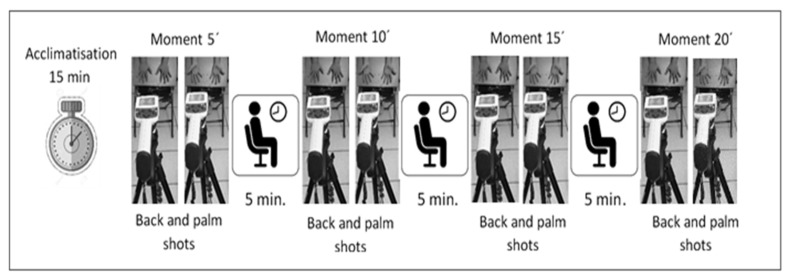
Experimental setup diagram.

Afterwards, a black board formed of foil wood with plastic laminate was placed on the chair, on which the shots were taken of the palms and backs of the hands. This board had tape markers, which worked as guides to provide precise and reproducible positioning of the hands. Each participant was instructed not to touch the board directly to prevent hand heat from being retained on the board and causing noise on the thermograms. The participant was asked to position him/herself behind the chair and bend down a little until his/her fingers were positioned over the marks. Then, a sequence of infrared images was taken, spaced every 5 min at times 5, 10, 15, and 20 (based on Vardasca, R., E. Francis, J. Ring, P. Plassmann, C.D. Jones, and J. Gabriel [47], and García, A. [48]) for each participant (Figure 3). After each thermal image of the palms and back of the hands was taken, the participant waited seated in another chair, while five minutes remained to continue with the next shot, until the four moments were completed. Thermal imaging sessions were held Monday through Friday from 3:30 to 4:30 pm (hours established by the company), with three participants per day.

**Figure 4:**
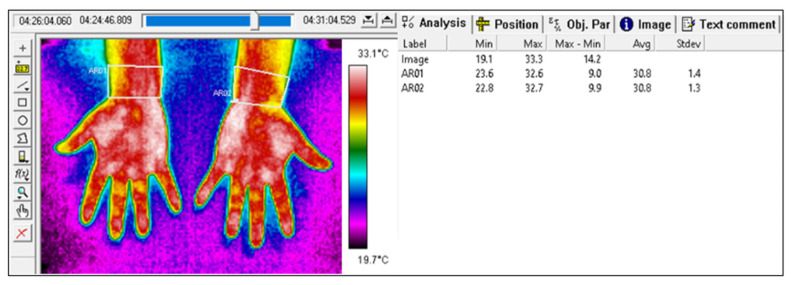
ThermaCAM Researcher Pro 2.10 software display.

The thermal images were then downloaded and analyzed through the ThermaCAM Researcher Pro 2.10 software, from FLIR Systems company, located at Boston, MA, USA, with which a total of 264 images were reviewed. When analyzing each IT image, the color palette was configured in the rain option. The emissivity (0.98) was already adjusted during the shots. The IR image was delimited according to the ROI in order to measure the temperature in that area. Then, the Results option was activated to display the temperature values of maximum, minimum, max-min, average, and standard deviation (Figure 4).

Next, the data were exported to Excel to organize and group according to the times in which the temperatures were recorded (5′, 10′, 15′, and 20′). Afterwards, the temperature differences were calculated for the minimum and maximum values of the temperature captured by the thermographic camera. Thereafter, the thermal asymmetries that could represent a possible injury were identified and classified in their levels of alarm and severity, as established by Marins et al. [49], and as shown in Table 1.

Subsequently, an OCRA evaluation was performed in the two study areas to compare the results with the proposed model.

#### 2.4.4. Statistical Analysis

For the analysis process of the acquired data, the study factors were grouped together. Age was grouped in young age (<40 years of age) and mature age (≥40 years old), and for gender, value 1 was assigned to male and 0 to female; BMI was classified as non-obese (<30 kg/m^2^) and obese (≥30 kg/m^2^). BP was classified in normal, high, and low, with normal and high heart rate, and RR in normal and high. Value 1 was assigned to subjects who had an injury or discomfort, and 0 to participants with no injuries.

The statistical analyses were carried out in IBM SPSS Statistics v.25^®^ software, from IBM Corp at Armonk, NY, USA, with which the data normality tests were performed using the Shapiro–Wilk test. Once the normal and non-normal data were identified, non-parametric tests were performed (Mann–Whitney), which were applied to all data due to the small sample size [50]. The relationship of the influence of the factors with the temperature differences was determined. Mixed-design analysis of variance was used to identify significant factors. Regression models and response surfaces were generated by Minitab 17. Three types of regression models were constructed using (a) study factors (age, gender, BMI, BP, RR, and heart rate), (b) relevant risk factors in OCRA (recovery factor, strength factor, posture and movement factor, and frequency factor), and (c) all of the above. The differences obtained with *p* < 0.05 were considered statistically significant with a 95% confidence interval.

The following flow chart summarizes the procedure carried out to obtain thermal images, up to the generation of the prediction models.

## 3. Results

### 3.1. Demographic Characteristics of the Participants

Table 2 and Table 3 show the series of data obtained by taking vital signs and somatometry pertaining to each of the participants in the sanding and tolex production areas, respectively.

### 3.2. Thermal Imaging

**Figure 5:**
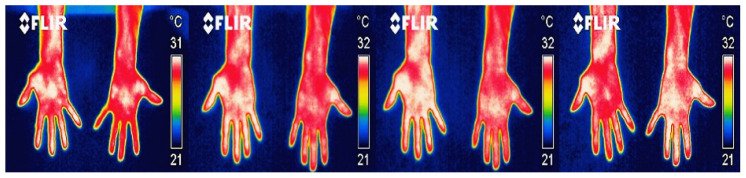
Example of thermogram taken from the front area of the palm at the times 5′, 10′, 15′, and 20′.

**Figure 6:**
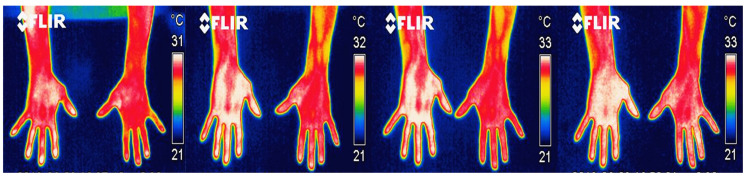
Thermogram from the back of the hand of one of the participants at moments 5′, 10′, 15′, and 20′.

A total of 264 thermal images from the palmar and dorsal regions of the hand were analyzed, nineteen from the sanding area and fourteen from the tolex area. Figure 5 and Figure 6 show some examples of the images captured from the palmar and dorsal regions of the hand, respectively. The thermograms were from moments 5′, 10′, 15′, and 20′ after they had performed their tasks for approximately 8.5 h, with two breaks (30 min for the first break and 15 min for the second).

The first thermogram was taken after 5 min of rest, and after the participant’s highly repetitive activity (in this case, sanding). The sequence of thermograms corresponds to a single participant. This volunteer presented the maximum asymmetry. In the images of Figure 5 and Figure 6, in particular, the fingers and wrist of the right hand show the highest temperature values compared to the left hand. However, this volunteer did not report having any injury or discomfort in his hands.

Table 4 shows the minimum and maximum temperature differences of the palmar and dorsal areas between the right and left wrists calculated for each period.

**Figure 7:**
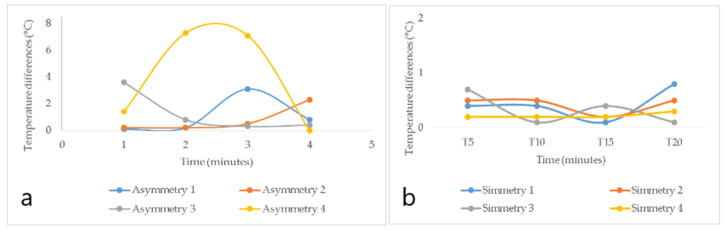
Behavioral graph of minimum temperature differences for the palm of the hand in the subjects of the sanding area. (**a**) corresponds to subjects with asymmetries. (**b**) represents subjects with symmetries.

**Figure 8:**
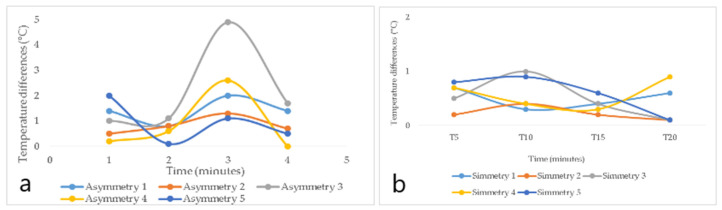
Graph of behavior of minimum temperature differences of the palm of the hand from the tolex area. (**a**) corresponds to subjects with asymmetries. (**b**) represents subjects with symmetries.

By analyzing the behavior of the minimum and maximum temperature differences of the participants’ wrists from their palmar and dorsal hand areas over time (5′, 10′, 15′, and 20′), the palmar zone showed higher temperature values than the dorsal region in the minimum temperature differences. This is true for both study areas, as shown in Figure 7 and Figure 8. In other research carried out by García, A., C. Camargo, J. Olguín, and J.A.L. Barreras [51], highly repetitive activities were analyzed by means of sensory thermography, in which wrist temperatures were evaluated, and, in all cases, temperature increases of more than 0.6 °C in 15 min were identified. In the same context, in the research of Camargo, C., J. Ordorica, E.J. De la Vega, J.E. Olguín, O.R. López, and J.A. López [52], where temperature changes in the wrists were measured by sensory thermography, when performing highly repetitive movements, the maximum temperatures of 35.078 °C in the right wrist and 34.663 °C in the left wrist were obtained.

Figure 7 shows the temperature difference behavior of the participants from the sanding area, and it was observed that four subjects had asymmetries (≥1.1 °C) on Figure 7a, representing the possibility of suffering from a CTD. Moreover, hands with asymmetry showed a great deal of temperature variations over time, while hands with symmetry (healthy hands) produced minimal temperature variations (Figure 7b).

Figure 8 shows the temperature difference behavior of the participants from the tolex area, and it was observed that five participants had asymmetries (≥1.1 °C), representing the possibility of suffering from a CTD. Subjects with asymmetry showed a great deal of temperature variations over time (Figure 8a), with a tendency to decrease slightly in time 10′, then increase in time 15′, and finally decrease in time 20′. Healthy subjects, on the other hand, had minimal variations over time (Figure 8b).

**Figure 9:**
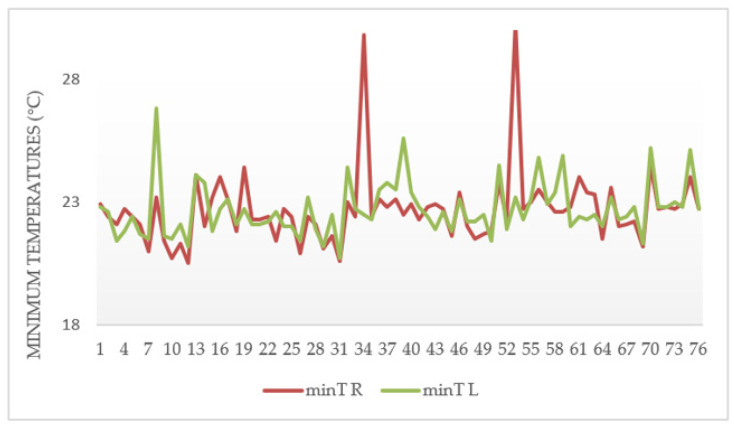
Graphs of minimum temperature differences between minT R (**right** wrist minimum temperature) and minT L (**left** wrist minimum temperature) points of subjects from the sanding area.

The minT R point represents the minimum temperature of the right wrist (palm side), and the minT L point corresponds to the minimum temperature of the left wrist (palm side). In Figure 9, there were fifteen cases with temperature difference between the minT R and minT L points representing thermal asymmetries (≥1.1 °C) with subjects probably suffering from a CTD.

**Figure 10:**
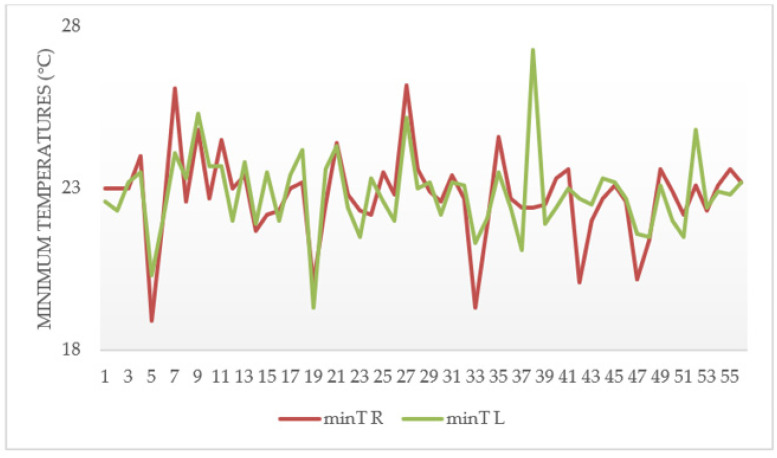
Graphs of minimum temperature differences between minT R (**right** wrist minimum temperature) and minT L (**left** wrist minimum temperature) points of subjects from the tolex area.

The minT R point represents the minimum temperature of the right wrist (palm side), and the minT L point corresponds to the minimum temperature of the left wrist (palm side). Ten cases of thermal asymmetries were observed in the graph of Figure 10, which represents subjects probably suffering from a CTD.

Regarding the diagnoses of injuries by means of an IT, Table 5 provides a summary of the participants who reported having symptoms with suspected CTD, or with a confirmed diagnosis of tendinitis, as well as the asymmetries obtained and their levels of attention and the diagnoses obtained by thermal imaging. It is worth pointing out that seven out of nine subjects were successfully classified. Table 6 shows the results of the OCRA evaluation to measure the risk of highly repetitive activities carried out in the study areas, where all of the participants are identified as having an unacceptably high risk, which coincides with the injury conditions of the participants shown.

**Figure 11:**
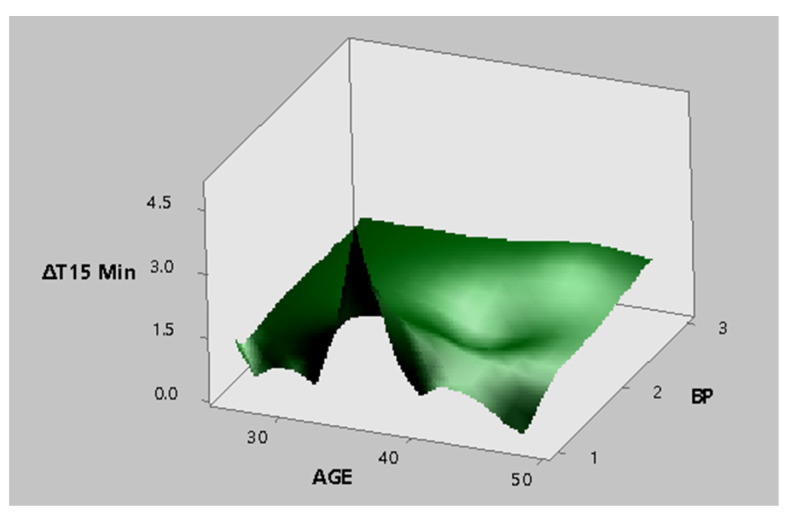
Response surface produced for the tolex area from the palm of the hand for the minimum temperature difference in time 15′ and the factors age and BP.

**Figure 12:**
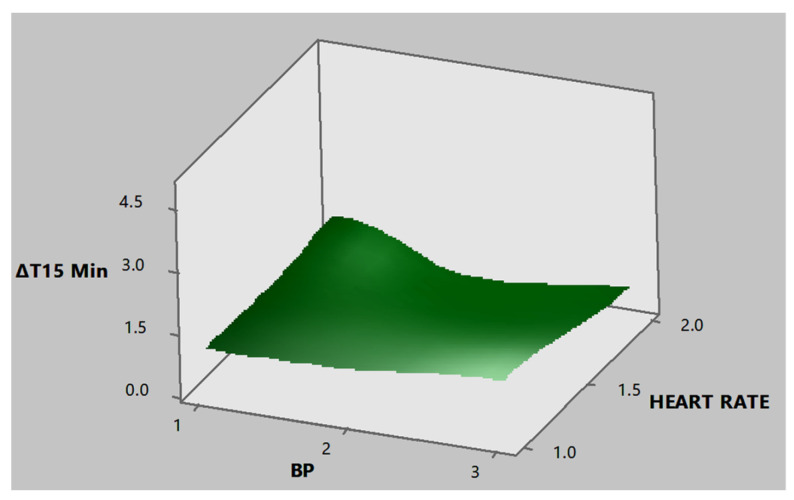
Response surface produced for the tolex area from the palm of the hand for the minimum temperature difference in time 15′ and the BP and heart rate factors.

According to the response surfaces generated, corresponding to Figure 11 and Figure 12, it was determined that the maximum values for temperature differences had normal levels in BP and heart rate.

The data normality test using Shapiro–Wilk yielded both normal and non-normal data. Since the two samples were small in size, the Mann–Whitney U test was performed for all data [50]. Table 7 shows a summary where the factors BP (*p* = 0.036, *p* = 0.014), heart rate (*p* = 0.047, *p* = 0.023), RR (*p* = 0.020, *p* = 0.036), and age (*p* = 0.010) were determined to be statistically significant for temperature differences, according to the non-parametric tests performed. The sanding area reported the factors BP, heart rate, and RR to be very significant, that is, associated with CTD, while in the tolex area, only the RR and age factors were determined as significantly linked with CTD. The sanding area showed higher significant values than the tolex area, predominating the time 10′ and the minimum temperature differences.

The mixed-design ANOVA results (see Table 8) confirmed a relationship between the factor BP (*p* = 0.009) and heart rate (*p* = 0.040) in the back, and BP (*p* = 0.009) and heart rate (*p* = 0.002) in the palm as significant regarding temperature difference in the sanding area. None of the factors evaluated were significant for the tolex area. Table 9 briefly shows the quadratic regression models generated according to the factors used. Firstly, the results of the study factors (age, gender, BMI, BP, RR, and heart rate) are displayed, with the determination coefficients, the estimates of which were adjusted appropriately to the study variable. Then, the values of the coefficients corresponding to the factors of the OCRA method are shown (recovery factor, strength factor, posture and movement factor, and frequency factor), and finally, we used all of the factors mentioned.

The highest values for temperature differences are observed in a middle age range (36 years) and with a normal level in BP.

## 4. Discussions

In this study, 75% of the cases that indicated injury or discomfort were detected by IT in the sanding area, while in the tolex area, just one case was reported. In addition, Papež et al. [53] and Papež, B., Jesenšek, M. Palfy, M. Mertik and Z. Turk [54] established that IT allows the correct classification of 72.2% of the hands, healthy and pathological, based on the dorsal part of the hand, while when seriously affected hands and healthy hands are evaluated, the percentage rises to >80%. Palfy and Papez [55] used 44 thermograms of healthy and pathological hands to determine the effectiveness of IT as a diagnostic method for CTS. Using IT and intelligence systems, they were able to diagnose cases of CTS with a success rate close to or above 80%.

Tkáčová et al. [20] recorded 14 thermal images to determine the level of effectiveness of IT for diagnosing CTS. The success rates found in the classification of healthy and pathological hands for were close to 80%. In the same context, Tchou et al. [56] recorded 122 thermograms, and obtained success rates for the classification of healthy hands with CTS pathologies close to or higher than 80%.

Therefore, IT has been shown to be remarkably effective in detecting asymmetries, which are known to be disease indicators, particularly CTS. However, our research detected asymmetries in the dorsal and palmar surfaces of the hand, contrary to other studies [20,21,53,54,55,56], who concluded that the dorsal side of the hand provides more satisfactory results when diagnosing CTS than the palmar area of the hand.

Ninety percent of the participants from the sanding area exhibited asymmetries with alert or serious attention. Out of the 17 subjects with asymmetries, 76% exhibited levels outside the normal range in at least one of the study factors (age, BMI, BP, heart rate, or RR), while 79% of the participants from the tolex area exhibited asymmetries with alert or serious levels. Out of the 11 subjects with asymmetries, 73% had levels outside the normal range in at least one of the study factors.

As for the sanding area, 94% of the participants with asymmetries are men (with less than one year working in the area), and the other 6% is the only female operator in the area. Regarding the tolex area, 36% of the participants with asymmetries are women, while 64% are men (the subjects have been working in the area for two to eight years.) It should be inferred that the sanding area would have a higher percentage of injuries, due to the fact that the production activities that are carried out demand more movement for the wrists than for the tolex area.

Moreover, other hypotheses are based on the production rate that has been increasing by 50%, leading to faster movements in the wrists, thus hurting them. Given that seven out of eight people with injuries from the sanding area have been working there for such a short time (less than a year), this is alarming. In fact, one operator who had been working in the area for only six months was disabled due to tendinitis during the study; this was contrary for the participants from the tolex area who have been working there for two to eight years. Another participant was also diagnosed with tendinitis, although it was not detected by IT.

By analyzing the behavior of the minimum and maximum temperature differences by time, the highest value of 6.5 °C (temperature difference between the back of the right and left hand) was identified for the sanding area in the minimum temperature values, in a time of 15 min for the back of the hand section. For the tolex area, the minimum temperature differences with the highest value on the back of the hand were shown in time 10′ (2.6 °C). For the maximum temperature differences, the highest value (1.6 °C) was recorded at time 15′. In the palms section, its maximum value in the sanding area was 7.3 °C for the minimum temperature difference between the right and left palm, recorded in time 10′. In the case of the tolex area, for the palmar region of the hand, the highest value was in time 15′with 5 °C for the minimum temperature difference. On the other hand, for the maximum temperature difference, its highest value was 1.1 °C in time 20′.

The factors age, BMI, heart rate, and RR have data with a normal distribution, whereas the factors gender, dominant hand, and BP exhibited abnormal distribution for the sanding area. In the tolex area, normality tests identified age and BMI as data with normal distribution, and the factors gender, BP, injury, heart rate, and RR exhibited abnormal distribution.

When carrying out the non-parametric tests for the back of the hand, the influence of the BP on the maximum temperature difference in time 10′ for the sanding area was verified. The relationship between the minimum temperature differences and BP for the palmar section of the hand was identified in time 10; heart rate in times 10′and 15′; and finally, RR in the minimum temperature difference in time 20′. An influence relationship between RR and the maximum temperature differences for the section of the back of the hand was found in time 15′. As for the tolex area, an influence relationship between RR and the maximum temperature differences for the back section of the hand was found in time 15′. There was no influence relationship for the palmar region of the hand.

The factors that influence the minimum temperature differences on the back of the hand for the sanding area are BP in time 15′, heart rate in times 10′ and 15′, and RR in time 20′. A relationship with BP in time 10′ was identified for the maximum temperature differences.

Regarding the non-parametric tests in the tolex area, in the section of the back of the hand, an influence relationship was found between RR and the maximum temperature differences in time 15′. In the palmar section of the hand, an influence was identified between age and the minimum temperature differences in time 10′. Concerning the factors that influence the minimum temperature differences in the palm of the hand for the sanding area, these are BP in time 10′, heart rate in times 10′ and 15′, and RR in time 20′.

The results indicate that the interaction between the significant factors for the sanding area and the main asymmetries had a low BP. On the other hand, in the tolex area, 80% of the asymmetries had normal BP. With regard to heart rate, for the palm of the hand in the sanding area, one out of three asymmetries were observed with a high heart rate (the highest value) for time 10′, while in time 15′, two out of three asymmetries exhibited a high heart rate. In the tolex area, 80% of the asymmetries included normal heart rhythms. For the RR, both areas were found to have normal RR for the diagnosed asymmetries. Regarding age, in the tolex area, 100% were young, both for time 10′. In contrast, other research [23,30,32,35] established that CTS increases with age, while several studies establish that age is associated with the prevalence of CTS [22,31,33,57].

In the area of sanding, it was not possible to evaluate the gender factor, since there is only one woman in this section. However, various studies [22,24,29,30,35] found an association between CTS and the female gender. However, there is also research that found no relationship between gender and CTS [27]. In the present study, BMI was not determined to be a significant risk factor, whereas other studies have found a strong association with CTS [22,30,33,35,57,58,59,60,61].

When carrying out the mixed-design analysis of variance for the sanding area, BP and heart rate turned out to be significant factors for the back and palm. Based on the mixed-design analysis of variance, for the tolex area, factors were determined to be non-significant. The regression models developed with the study factors obtained a coefficient of determination of 0.9737 for the sanding palm area, and a coefficient of determination of 0.9667 for the tolex palm area. It is worth mentioning that, to attain these percentages, all of the study factors are included in the equations, since omitting them would considerably reduce the determination coefficients.

On the other hand, the models constructed using the OCRA method generated lower coefficients of determination. For sanding palm, R^2^ = 0.5854 and for tolex palm, R^2^ = 0.3481. However, when both groups of factors were integrated into a single regression model, the best results were obtained with the following coefficients of determination: 0.9850 for sanding palm and 0.9871 for tolex palm. Meanwhile, research conducted by Márquez Gómez, M. [41] achieved an accuracy of 83.91% and a kappa index of 63.14% in their statistical model for the prediction of WRMSD-related (work-related musculoskeletal disorders) discomfort in the hands/wrists, in which they considered six factors: postural overload, repetitiveness of movements, gender, medical history related to MSD, frequency of household chores, and job rotation. In another study, multiple regression models were developed to predict the combined frequency and severity of the pain from WRMSDs, achieving an R-squared of 32.9% [62]. In addition, Sasikumar, V. and S. Binoosh [63] employed various machine learning algorithms in their predictive model for WRMSDs among computer professionals, considering postural, physiological, and work-related factors, with an accuracy of 81.25%.

## 5. Limitations

During the process of obtaining data in a maquiladora* company and acquiring the thermograms, the humidity levels (45% to 60%) depended greatly on the climatic conditions. For the application of questionnaires and the taking of vital signs and somatometry, as well as the taking of thermal images, there was a restricted schedule in order not to affect the daily production goals for the company.

## 6. Conclusions

This research proved the effectiveness of IT in the detection of suspected CTS injuries by means of thermal asymmetries in the wrists. Three temperature prediction models were developed based on the study factors, two for the sanding area, with which it is possible to make predictions of the temperature differences for times of 10 and 15 min to determine whether or not the subject exhibits thermal asymmetries that could lead to injury, and another for the tolex area, with predictions for times of 15 min. The maximum thermal asymmetries were registered for the minimum temperature differences, both in the sections of the back of the hand and in the palm of the hand in both study areas.

Ninety-four percent of the participants in the sanding area had thermal asymmetries with a degree of alert or seriousness attention, while for the tolex area, only 73 percent had them. In the tolex area, 36% of the participants with thermal asymmetries with a degree of alert or seriousness attention are women, while 64% are men. The subjects have been working in this area from two to eight years. In the area of sanding, 94% of the participants with asymmetries (alert or seriousness level) are men, and have been working in the area for less than one year.

Factors such as age, BP, heart rate, and RR were found to highly influence injuries in participants. However, no strong association with BMI was found. It is inferred that participants who exhibit levels out of the normal range for at least one of the factors, such as advanced age (≥40 years), BMI, BP, heart rate, or RR, are highly likely to have wrist injuries with a level of attention that is alarming or serious, so it is important to follow up with staff who exhibit such characteristics and consider these results during the recruitment process. In this study, in sanding area, the RR factor stands out, with a high level in personnel with thermal asymmetries of alert or seriousness, while for the tolex area, advanced age predominates.

Finally, these are preliminary results from a limited number of thermal images, so in the next section proposals are made that include increasing the sample size.

## 7. Future Research

To continue with the development of this research, it is proposed to carry out future studies of wrist temperature, comparing temperatures before starting activities with the peak time of work activity. This will allow for a more accurate assessment of temperature differences compared to time. It is suggested to analyze the values of average temperatures, as well as the study of other ROIs in the hands. In addition, it is recommended to carry out studies of temperature differences considering a large sample size, where there is a greater number of participants of both genders, and, if possible, with dominant hand variation. Only nine subjects with injuries were reported, and in one of the study areas, only one person had a left dominant hand, while there were none in the other. For this reason, it is advisable to extend the investigations to other production areas of the same company. In turn, it is advisable to analyze other factors particular to each process, such as force, vibrations, movement amplitude, postural overload, length of employment, overtime, and rotation, or, in the case of tolex, carry out studies on the cold factor, since this area works with low temperatures. Furthermore, it is proposed to apply the standardized Cornell Musculoskeletal Discomfort Questionnaire (CMDQ), used by Acquah et al. [64] in their study of the prevalence and intensity of symptoms of musculoskeletal disorders (MSDs) in workers of informal recycling of electrical and electronic waste. In addition, it is suggested to consider demographic factors, such as smoking, MSD-related medical history, exercise, household chores (studied by Márquez Gómez, M. [41]), physiological factors, work-related factors [63], job fatigue, and strain [65]. Furthermore, we recommend future research on the development of regression models with acceptable determination coefficients, in which only the found influence factors are included. At the same time, we propose to use the machine learning method for the development of predictive models and to make comparisons between both results. Finally, it is suggested to extend the study to other upper extremities, lower extremities, and the lower and upper back to analyze the behavior of temperature differences and to make comparisons between these.

## Figures and Tables

**Table 1:** Scale of the level of attention given according to differences of temperatures between the body ROI (body region of interest) against laterals or between two shots of the same ROI.

Temperature Differences	Level of Attention
≤0.4 °C	Normal
0.5–0.7 °C	Follow-up
0.8–1 °C	Prevention
1.1–1.5 °C	Warning
≥1.6 °C	Seriousness

Source: Marins, J. C. B., I. Fernández, J. Arnaiz, A. A. Fernandes and M. Sillero [49].

**Table 2:** Demographic characteristics from the sanding area.

Attribute	Category	QTY	Percentage
Gender	Male	18	95%
	Female	1	5%
Age	Average = 33 ± 9.7 years		
	Rank = 18–52 years		
BMI	No obese	14	74%
	Obese	5	26%
	Average = 27 kg/m^2^		
BP	Normal	16	84%
	Low	1	5%
	High	2	11%
Heart rate	Normal	17	89%
	High	2	11%
RR	Normal	11	58%
	High	8	42%
Injury	Eight (men)		
Dominant hand	One left-handed	person	

QTY (quantity), BMI (body mass index), BP (blood pressure), RR (respiratory rate).

**Table 3:** Demographic characteristics from the tolex area.

Attribute	Category	QTY	Percentage
Gender	Male	8	57%
	Female	6	43%
Age	Average = 35 ± 7.45 years		
	Rank = 26–48 years		
BMI	No obese	9	64%
	Obese	5	36%
	Average = 28.4 kg/m^2^		
BP	Normal	11	79%
	Low	1	7%
	High	2	14%
Heart rate	Normal	10	71%
	High	4	29%
RR	Normal	10	71%
	High	4	29%
Injury	One (woman)		
Dominant hand	100% right-handed		

**Table 4:** Values of the minimum and maximum temperature differences from the back of the hand obtained from the participants by time.

Participant	ΔT5 Min	ΔT5 Max	ΔT10 Min	ΔT10 Max	ΔT15 Min	ΔT15 Max	ΔT20 Min	ΔT20 Max
Subject 1 with an injury	0.3	0.4	2.1	0.4	0.2	0.7	0.3	0.7
Subject 2 with an injury	0.5	0.3	1.6	0.2	0.2	0.4	0.1	0
Healthy Subject 1	0.4	0	0.6	0.4	0.8	0.4	1.6	0.2
Healthy Subject 2	3.4	0.6	1	0.5	2.9	0.1	2.3	0.2
Healthy Subject 3	0	0.4	0.4	0.1	0.5	0.2	0.2	0.1

ΔT = The temperature (minimum or maximum) of the back of the right hand and the temperature (minimum or maximum) of the back of the left hand.

**Table 5:** Diagnostic results of asymmetric wrist injuries in the palmar region of the hand.

Subject with Injuries	Area	Maximum Asymmetry (°C)	Level of Attention	Injury Diagnosis (Warning/Seriousness)
1	Sanding	3.1	Serious	Yes
2	Sanding	2.3	Serious	Yes
3	Sanding	2.1	Serious	Yes
4	Sanding	0.9	Prevention	No
5	Sanding	1.8	Serious	Yes
6	Sanding	2	Serious	Yes
7	Sanding	0.4	Normal	No
8	Sanding	1.7	Serious	Yes
9	Tolex	4.9	Serious	Yes

**Table 6:** Results of the OCRA assessment conducted in the study areas.

Subject with Injuries	Area	OCRA Checklist Index	Risk Level	Recommended Action	Equivalent OCRA Index
1	Sanding	63	unacceptable high risk	Job upgrading, medical supervision, and training	>9
2	Sanding	63	unacceptable high risk	Job upgrading, medical supervision, and training	>9
3	Sanding	63	unacceptable high risk	Job upgrading, medical supervision, and training	>9
4	Sanding	63	unacceptable high risk	Job upgrading, medical supervision, and training	>9
5	Sanding	63	unacceptable high risk	Job upgrading, medical supervision, and training	>9
6	Sanding	63	unacceptable high risk	Job upgrading, medical supervision, and training	>9
7	Sanding	63	unacceptable high risk	Job upgrading, medical supervision, and training	>9
8	Sanding	63	unacceptable high risk	Job upgrading, medical supervision, and training	>9
9	Tolex	51.8	unacceptable high risk	Job upgrading, medical supervision, and training	>9

**Table 7:** Mann–Whitney U-test of study factors for temperature differences.

Area-Section	Factor	Time (Min)	Temperature Differences	*p*-Value
Sanding-Back	BP	10	Maximum	0.036
Sanding-Back	BP	15	Minimum	0.014
Sanding-Palm	BP	10	Minimum	0.014
Sanding-Palm	Heart rate	10	Minimum	0.047
Sanding-Palm	Heart rate	15	Minimum	0.023
Sanding-Palm	RR	20	Minimum	0.02
Tolex-Back	RR	15	Maximum	0.036
Tolex- Back	Age	10	Minimum	0.01

**Table 8:** Mixed-design analysis of variance of the study factors for the sanding area.

Area-Section	Factor	*p*-Value
Sanding-Back	BP	0.009
Sanding-Back	Heart rate	0.040
Sanding-Palm	BP	0.009
Sanding-Palm	Heart rate	0.002

**Table 9:** Summary of models by zone, hand section, and factors included.

Area	Hand Section	Model (Factors Included)	R-sq	R-sq (Adj)
Sanding	Back	Proposed factors	0.8836	0.7905
Sanding	Palm	Proposed factors	0.9737	0.9475
Tolex	Palm	Proposed factors	0.9667	0.9134
Sanding	Back	OCRA factors	0.2583	0.0993
Sanding	Palm	OCRA factors	0.5854	0.4965
Tolex	Back	OCRA factors	0.3455	0.1492
Tolex	Palm	OCRA factors	0.3481	0.1525
Sanding	Back	All factors	0.9078	0.6864
Sanding	Palm	All factors	0.9850	0.9488
Tolex	Back	All factors	0.9697	0.8031
Tolex	Palm	All factors	0.9871	0.9164

R-sq (coefficient of determination). R-sq (Adj) (Adjusted coefficient of determination).

## Data Availability

Written informed consent has been obtained from the participant(s) to publish this paper.
